# *SurfaceSlide*: A Multitouch Digital Pathology Platform

**DOI:** 10.1371/journal.pone.0030783

**Published:** 2012-01-23

**Authors:** Yinhai Wang, Kate E. Williamson, Paul J. Kelly, Jacqueline A. James, Peter W. Hamilton

**Affiliations:** 1 Centre for Cancer Research and Cell Biology, Queen's University Belfast, Belfast, United Kingdom; 2 Department of Pathology, Royal Victoria Hospital, Belfast, United Kingdom; University of Vermont, United States of America

## Abstract

**Background:**

Digital pathology provides a digital environment for the management and interpretation of pathological images and associated data. It is becoming increasing popular to use modern computer based tools and applications in pathological education, tissue based research and clinical diagnosis. Uptake of this new technology is stymied by its single user orientation and its prerequisite and cumbersome combination of mouse and keyboard for navigation and annotation.

**Methodology:**

In this study we developed *SurfaceSlide*, a dedicated viewing platform which enables the navigation and annotation of gigapixel digitised pathological images using fingertip touch. *SurfaceSlide* was developed using the Microsoft Surface, a 30 inch multitouch tabletop computing platform. *SurfaceSlide* users can perform direct panning and zooming operations on digitised slide images. These images are downloaded onto the Microsoft Surface platform from a remote server on-demand. Users can also draw annotations and key in texts using an on-screen virtual keyboard. We also developed a smart caching protocol which caches the surrounding regions of a field of view in multi-resolutions thus providing a smooth and vivid user experience and reducing the delay for image downloading from the internet. We compared the usability of *SurfaceSlide* against Aperio ImageScope and PathXL online viewer.

**Conclusion:**

*SurfaceSlide* is intuitive, fast and easy to use. *SurfaceSlide* represents the most direct, effective and intimate human–digital slide interaction experience. It is expected that *SurfaceSlide* will significantly enhance digital pathology tools and applications in education and clinical practice.

## Introduction

Pathology sits at the core of diagnostic cancer care in the UK National Health Service (NHS) and informs treatment and management of disease in healthcare globally. Typically tissue and cellular samples prepared on glass slides are visually examined by an experienced pathologist using a microscope to identify the morphological features and patterns which inform the pathological diagnosis. Although the knowledge underpinning pathology has advanced significantly in recent years, the technology lags behind with most pathologists still making their diagnoses using conventional microscopy and visual assessment. This practice, in existence for 150 years, is on the verge of change.

Digital pathology represents a step shift change in pathology by providing a digital environment for the management and interpretation of pathological images together with their associated data [1]. This has been made possible by the development of scanning devices that can digitally scan Whole Slide Images (WSIs), *aka*. digital slides or virtual slides, at diagnostic resolution (0.25 µm per pixel) [2]. Indeed, some scanners are able to scan at higher resolution of 100× magnification (0.14 µm/pixel) using oil immersion. Although the images generated are unwieldy, the use of these digitised slides is increasing in a range of diagnostic pathology applications. The scanned images of typical tissue specimens can exceed 120,000×80,000 pixels in size (i.e. or 28 gigabytes of uncompressed data [3]). These can subsequently be viewed and annotated on computer monitors, using a combination of mouse and keyboard controls.

Digital pathology is impacting on pathology education [4], [5], [6], assessment and Continuing Professional Development (CPD) [4], [7], and is facilitating tissue-based research [8], [9], [10] and enhancing clinical practice [11]. In education, digital slide visualisation has been enhanced using the “Powerwall” [12], [13], a multiple screen configuration which allows digital slides to be viewed and navigated in high resolution on a wall sized space. Research applications have focussed on the analysis of digital slide images and developing pattern recognition software for the understanding and image data [10], [14], [15], [16] as well as decision support systems for diagnosis and prognosis [17], [18]. Due the ultra-large size of digital slides, research is also focused in the area of high performance computing [19], [20], [21] for the high throughput analysis of digital slide imageries. In routine clinical diagnosis, digital pathology is beginning to have a more defined role in remote consultation/second opinion scenarios, through online sharing of digital slides [21].

While digital pathology and slide scanning techniques continue to develop, a number of technical and behavioural challenges remain and there is considerable scope for improving the interface and methods for human–digital slide interaction. Unlike the traditional microscope, the interaction, i.e. viewing and annotation of digital slides, is achieved using a combination of mouse and keyboard which is not intuitive. Further, the contact between the user and the digital slide is indirect which has been shown to slow down the time it takes to interpret a digital slide, and has impeded the acceptance of the digital pathology technology.

Multitouch refers to the technology that enables our interaction with virtual objects through the use of hardware and software to recognize, track and interpret multiple simultaneous touches on a touch screen [22]. Multitouch emanates from our well developed skills, such as flick and grasp, for physical object manipulation [23]. It represents a much more intuitive way to find what you want and to learn how to use the commands. Targets of actions can be quickly achieved using only a few commands which the user executes in an unmediated fashion. It is commonly known as Natural User Interface (NUI). There are a few reported prototype multitouch systems [24], [25], [26], [27], [28], however none of them are designed for pathological studies, and it would require a significant amount of work to reproduce their hardware and to design digital pathology platforms on them.

Digital slides are ultra-large in size and need to be served out using Region-on-Demand approaches. Using the software developments described in this manuscript the entire digital slide image does not need to be downloaded to the client computer; only the region/resolution that is currently being viewed. This can be implemented by loading and displaying a screen sized Field of View (FoV) at runtime. However FoV loading delay may occur using this approach, which is attributable to the indexing of FoVs (from digital slides), network latency and image decompression.

The aim of this project was to investigate how multitouch technology could be utilised in digital pathology to develop an enhanced human–digital slide interaction interface which enabled people to view and annotate online digital slides intuitively using their finger tips.

## Materials and Methods

### *A*. A. Multitouch and Microsoft Surface

**Figure 1:**
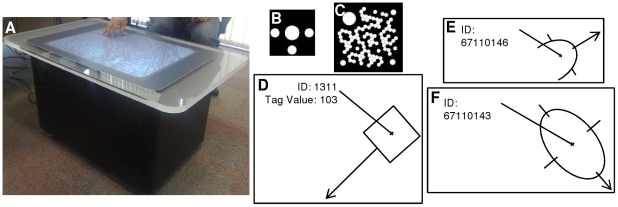
Microsoft Surface machine and the three types of objects which it can recognise. (A) Microsoft Surface tabletop computer, (B) a byte tag object (8 bits), (C) an identity tag object (128 bits), (D) a recognised tag object on Microsoft Surface, (E) a recognised finger on Microsoft Surface, (F) a recognised general blob on Microsoft Surface. *Figure D, E and F are redrawn images generated using Input Visualizer software from Microsoft.

We selected the commercially available Microsoft Surface as a vehicle to develop multitouch technology in pathology and to overcome a number of technical design problems. The unique vision system provided by Microsoft Surface has the ability to recognise and track the movements of three types of objects: blobs, tags, and finger touches. Two examples of tags are shown in Figure 1B–1C. Each individual tag has a unique tag value, which can be visualised using Microsoft Surface Input Visualizer as a square with its associated tag value and orientation (as the arrow indicates in Figure 1D). Finger touch also has a unique ID and orientation (Figure 1E). Objects other rather than tags and fingers are recognised as a general blob (Figure 1F). Microsoft Surface is able to recognise up to 52 simultaneous finger touches, which provides the opportunity to develop multi-user software applications for multi-participant engagement (e.g. 5 people sitting around it). It terms of the manipulation of digital slides, Microsoft Surface makes it possible for not only a single user to perform a number of tasks, such as the navigation and annotation of digital slides, but also for multiple users to engage in the discussion and manipulation of a digital slide.

#### Hardware

The Microsoft Surface is 30-inch tabletop computer (Figure 1A) with a vision system enabled by 5 embedded short-range cameras which collect and deliver camera images for input analysis. It uses a 2.13-GHz Intel Core 2 Duo processor and supports connection to the Internet via both wired and wireless LAN. Its four major components, comprising a high-specification computer, camera system, light engine and tabletop are volume reduced within the table [29].

**Software**: The development of software applications using Microsoft Surface is well supported by Microsoft. It is installed with a Windows Vista operating system. A dedicated Surface software development kit (SDK) is provided for the design of applications. Users are able to develop applications using. NET Framework, Microsoft Visual Studio (C# language), Microsoft Expression Blend 2, Windows Presentation Foundation (WPF) and Extensible Application Markup Language (XMAL).

### *B*. B. PathXL i-Server Interface

Digital slide images were hosted on PathXL's remote load-balanced image server cluster, from which *SurfaceSlide* had access via the Internet. Digital slides were stored in a multi-tile, multi-resolution format. Due to their size, digital slides were served using a *Tile-on-Demand* method that downloads only the regions of interest (ROIs) onto the client computer. The PathXL i-Server is configured to manage multiple requests from the client viewer and to serve out the appropriate regions of the image at the requested resolution. Its recognised instructions are listed in Table 1. An interface between the Microsoft Surface and the PathXL i-Server allowed us to configure a dedicated Surface viewer for navigating digital slides on the Microsoft Surface instrument.

**Table 1:** PathXL i-Server Instructions.

Instructions	Explanations
**x**	the x coordinate of the upper left corner of the ROI
**y**	the x coordinate of the upper left corner of the ROI
**width**	width of ROI (in pixels)
**height**	height of ROI (in pixels)
**zoom**	the magnification of ROI to be loaded, the value is in the range of [1,∞] where 1 indicates the highest magnification.

### *C*. C. Methods

**Figure 2:**
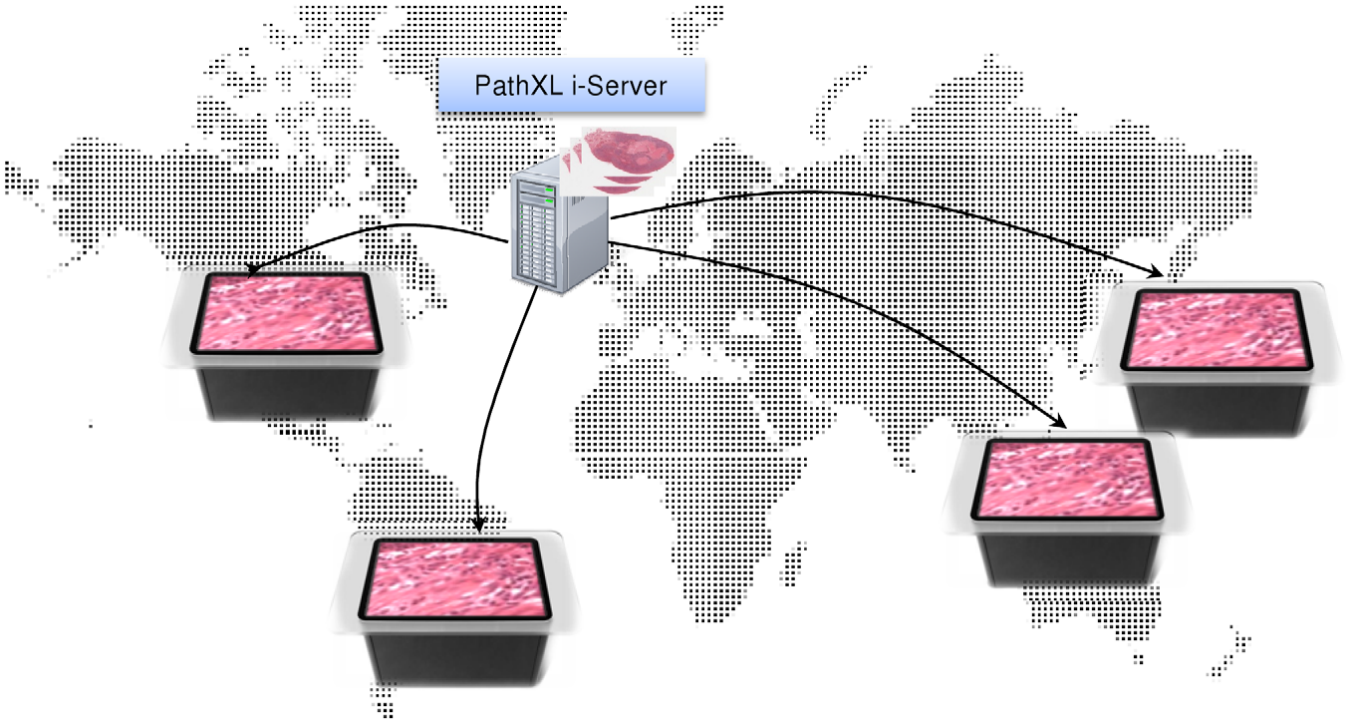
*SurfaceSlide* design principle, to allow the access of a same slide from different sites.

The aim of this project was to develop an effective human–digital slide interaction platform with coincident user perception and action spaces. A single user should be able to easily control the navigation of digital slides, to locate a single digital slide (from a collection of slides) and view any field at any magnification. The coffee table shaped Microsoft Surface provides a perfect platform for a small group of users to sit around for discussion. Consequently it is necessary that the design of *SurfaceSlide* should be suitable for multi-user interactions. It requires not only that the information displayed on-screen is orientation invariant, meaning that a user, wherever he/she is sitting, can view images right side up, but also that multiple users can interact simultaneously. Further it should also accommodate the access requests from many sets of Microsoft Surface. This design could have significant implications across many aspects of pathology, such as pathology education and group consultation regardless of the physical location of the participants (Figure 2).

**Figure 3:**
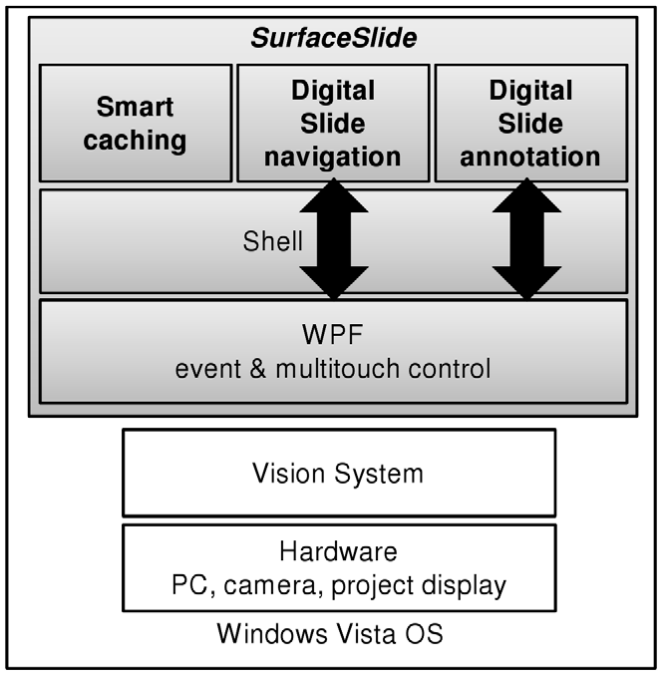
*SurfaceSlide* software architecture. Three functionality modules, designed using Microsoft Surface, were digital slide navigation, digital slide annotation and smart caching.

*SurfaceSlide* was programmed using Microsoft Visual Studio and Surface SDK, with the programming language of C# and WPF. Three functional modules were specifically engineered and encapsulated in the forms of C# classes for reusability, including digital slide navigation, digital slide annotation and smart caching. As shown in Figure 3, these three modules sit at the top of *SurfaceSlide* application layer. The digital slide navigation and annotation modules were designed using WPF for the control of multitouch actions and event handling. The Vision System layer consists of device drivers, digital signal processing unit, object recognition and other functional infrastructure, which directly communicates with the embedded hardware.

The digital slide navigation module controls panning and zooming in and out operations for the viewing of digital slides. The digital slide annotation module controls the logics for creation and placement of annotations, which can be circles, rectangles or free style text created using an on-screen virtual keyboard. The smart caching module caches digital slide images in the background for a smooth and vivid slide viewing experience.

### *1)* 1) Digital Slide Navigation

In order to provide a sound basis for the development of *SurfaceSlide*, the types of image viewing tasks were firstly defined. The most common image navigation controls are panning and zooming operations. Panning moves the current FoV whilst zooming changes the magnification.

The digital slide panning and zooming operations are then assigned to corresponding gestures. Microsoft Surface SDK provides a 2D affine manipulation processor, which is able to track the amount of translation and scaling affine transforms. The panning operation of digital slides can be performed using 2D translation affine transforms whereas the zooming of digital slides can be simulated as 2D scaling affine transforms. As defined in the Surface SDK, by placing fingers on top of the touch screen, if one or more fingers move towards one direction, the amount of image translation occurred is recorded, whereas if two or more fingers are moving towards or apart from a centre of manipulation, the amount of image scaling is recorded. This provides enhanced flexibility in image manipulation which accommodates scaling transformations at any arbitrary location (not necessarily the centre) on the tabletop. However currently, zooming in/out is always performed towards the centre of the FoVs using e.g. the mouse scroll button. For Microsoft Surface, the scaling operation at a random location 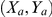 can be decomposed into a sequence of affine operations:
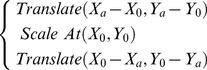
(1)where 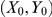 is the coordinates of the centre 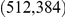 of the tabletop.

Users are also able to perform translation and scaling operations simultaneously where the amount of translation and scaling can be recorded at runtime. This feature significantly speeds up the rapid localisation of ROIs, and as far as the authors are aware, there are not any digital slide viewers able to perform this function using traditional mouse and keyboard. The recorded amount of translation and scaling factors are processed in the form of matrixes. Given a FoV 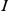, which is downloaded from PathXL i-Server, to be manipulated by a sequence of 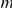 affine transformation 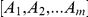 which could be translation or scaling operation, the transformed FoV 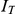 can be expressed as the product of the individual affine transforms applied on FoV 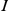.
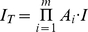
(2)


**Figure 4:**
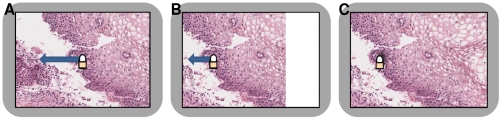
An illustration of how panning works on *SurfaceSlide* using a cervical tissue digital slide. (A) A left-panning operation is going to be applied on Microsoft Surface tabletop, (B) the on-screen image is moved to the left. Please note some regions are moved out of the screen on the left, whereas a white rectangle appears from the right, (C) The left-panning operation finishes as the finger lifts up, and a new FoV is loaded from the PathXL i-Server.

In order to show continuous image transformations while the on-screen image is being manipulated, intermediate affine transformation results 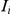 are calculated using:
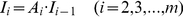
(3)



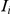
 are displayed rapidly and continuously at runtime until the whole sequence of affine transforms finishes which is also the moment all fingers leave the tabletop. A translation operation will move certain regions of the on-screen image out of the boundaries of the screen and displays white spaces. A zooming-in operation would also display white spaces, whereas a zooming-out operation could cause blur artefacts. This issue is addressed by recording the location of 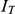 and replacing it by high resolution images loaded from PathXL i-Server. This process is illustrated in Figure 4.

**Figure 5:**
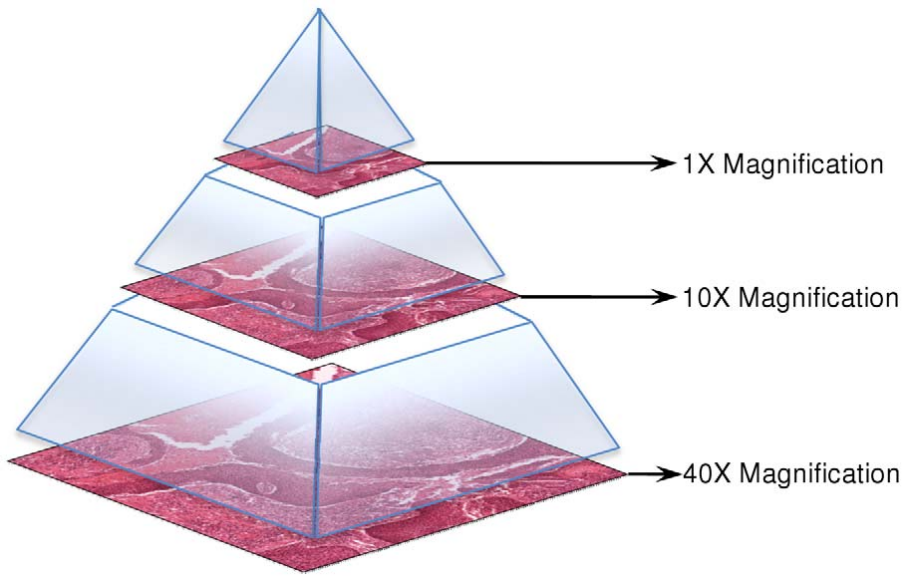
An illustration of how digital slides are stored in a pyramid structure. In this example, the highest magnification (40×) digital slide is stored. Two intermediate magnification (1× and 10×) slides are also stored in the pyramid.

When an image loading instruction (table 1) is sent to the PathXL i-Server, the server returns the image data with requested magnification. Digital slide images are stored on PathXL i-Server in a pyramid fashion as shown in Figure 5. For a single digital slide, a highest magnification (e.g. 40×) and a number of intermediate magnification images (e.g. 1× and 10×) are also stored in the pyramid. When an image loading instruction requests non-standard magnifications, such as 5.4× magnification images, its immediate higher magnification neighbour (e.g. 10× magnification image) is obtained followed by nearest neighbour interpolation to reduce it to 5.4× magnification. In this way, any non-existing magnification below the highest scanned magnification (40×) can be requested and displayed.

### *2)* 2) Digital Slide Annotation

**Figure 6:**
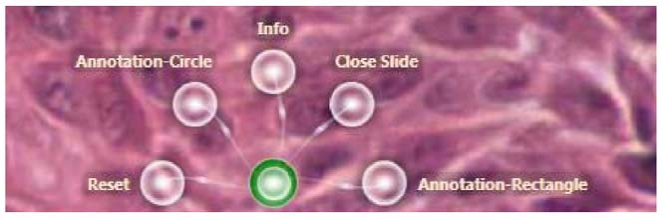
An example of menu elements on Microsoft Surface. The green circle is the menu icon. When it receives a finger touch, the five semi-transparent white menu elements appear indicating the different instructions which can be performed.

Digital slide annotation refers to the ability to draw shapes, such as a circle and a rectangle, on top of digital slide to mark ROIs, and to write free style text labels as descriptors of the defined area. The conflicting needs of digital slide navigation and annotation drawing represent one of the engineering challenges. Traditionally, users are able to use a pen to mark on a glass slide, or for digital slides, to identify a specific annotation command from a menu bar using mouse clicks. For a multitouch application, it is possible to use specific gestures, such as placing your palm on screen, to trigger a digital slide annotation event. However, these are not intuitive hand gestures without training, and they can also be wrongly triggered. As a compromise, it was necessary to introduce a menu system to trigger annotations. We used a newly introduced menu element from the Surface SDK, as shown in Figure 6. The green circle is the menu itself. When it is touched using one finger, a list of menu elements appear.

*SurfaceSlide* implements two types of annotations: a circle and a rectangle. It also allows users to write text labels below the annotations using a virtual keyboard. The two types of the annotations and text labels are all wrapped in an Annotation Class. Certainly many rich annotation functionalities could be added, such as measuring tools and free style shapes. In this study, we implemented circles and rectangles to explore a number of usability issues relating to the adoption of multitouch technology in pathology.

**Figure 7:**
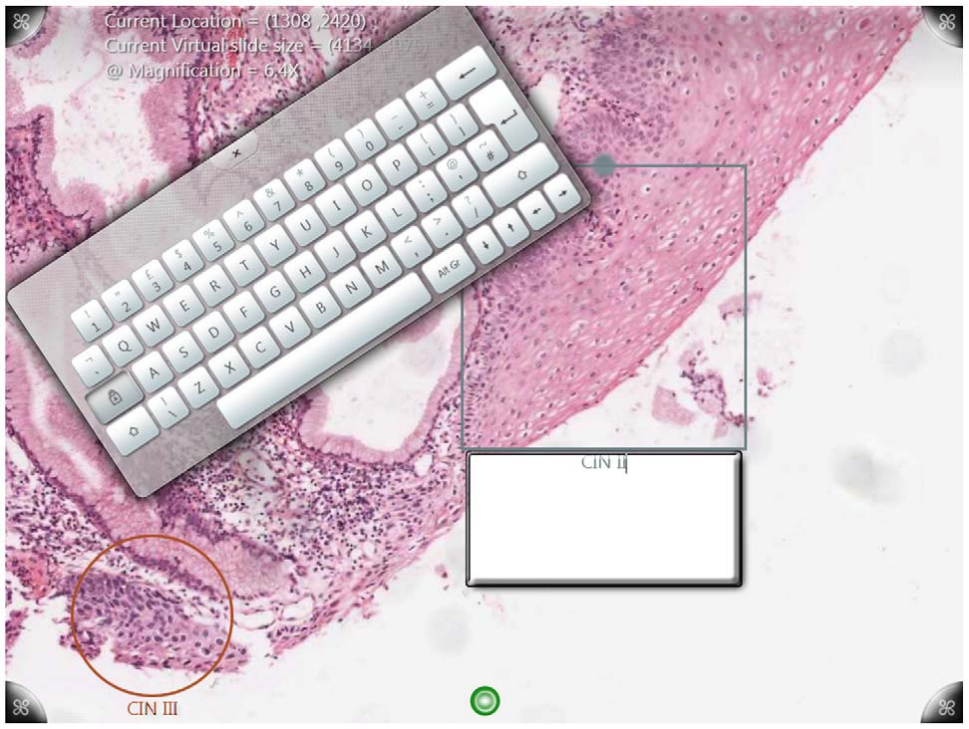
A screenshot of a cervical tissue digital slide during annotation. This cervical tissue digital slide is 29,762×34242 pixels in size. The region on the bottom right has been annotated with a circle and marked with text “CIN III” (Cervical Intraepithelial Neoplasia III), whereas the region in the middle is being annotated with a gray rectangular box and text “CIN II” (Cervical Intraepithelial Neoplasia II). Users are able to type in text annotations using the on-screen virtual keyboard.

When either the *Annotation-Circle* or the *Annotation-Rectangle* menu element (Figure 6) is touched, the gesture control to the digital slide is halted, and a new instance (object) of the Annotation Class is created. A colour coded thin-edge circle or rectangle appears on the tabletop. This instance comes with its own 2D manipulation processor which is able to recognise and react to touch events. Users are able to move and resize annotations with finger touches in a similar way to the manipulation of digital slides. When the corresponding textbox is touched, a virtual QWERTY keyboard appears on screen allowing text input (Figure 7). Texts inputs are stored in the form of a string in the annotation instances. The annotation process finishes when the *Enter* key is pressed on the virtual keyboard, meanwhile the manipulation of digital slides is restored.

The *xy*-coordinates of the centroid of the annotation are recorded, and the annotations are fixed within the digital slide. When the digital slide is panned and zoomed in and out, the system checks all existing objects, including the digital slide and all annotations. If there are a number of annotations, the same amount of panning and zooming operations will be performed for each annotation individually. Visually, it appears that the digital slides and all annotations are bonded and move together.

### *3)* 3) Smart Caching

The real-time viewing of digital slides with minimal image download latency is extremely important for the adoption and use of digital slides in pathology and ensures safety in diagnostic practice. However the speed of viewing using image data hosted on a remote server can be affected by many factors, such as network bandwidth, the amount of network traffic, the speed both to locate ROIs on the server and to decompress the image on the client machines. All the issues could cause delays for image downloading, and subsequently affect the smoothness of digital slide viewing. Many online image viewing applications experience similar problems, such as the well known Google Maps and Microsoft Bing Maps.

Therefore, we propose a smart caching method to eliminate or at least reduce the amount of white backgrounds when a digital slide is manipulated. The smart caching is driven from the client side viewer and implemented within multitouch *SurfaceSlide*.

The screen size of Microsoft Surface is 1024×768 pixels. Experiments suggest that to the human vision system it appears faster to load 12 256×256 pixel blocks instead of a whole 1024×768 pixel region. This phenomenon can be explained in the following way. If an instruction is triggered to load a whole 1024×768 pixel region, the computer will wait until image data from this 1024×768 pixel region is gathered in the physical memory before displaying it on screen. Hence a delay is expected. However, if the whole region is broken down to 12 256×256 pixel blocks, these 12 blocks will be displayed on screen asynchronously, as each block will be displayed once loading of its small amount of image data is completed. Therefore little delay is expected. In addition, the human eye will probably compensate for the delay of some of the 256×256 pixel blocks by imagining the continuous nature of tissue imageries. For this reason, a 3×4 grid was built to accommodate these 12 image blocks on screen.

**Figure 8:**
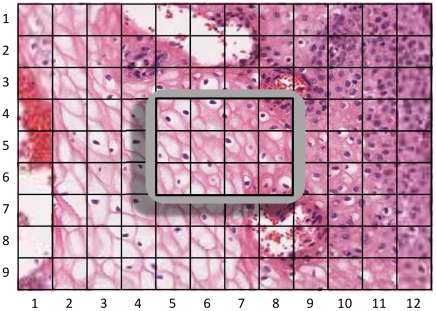
An illustration of smart caching. The gray rectangle in the middle of the image indicates the Microsoft Surface table. The grids in row 4–6 and column 5–8 are the 12 grids shown on screen. The rest of the grids are all cached in the background. Some of the cached grids are designed to use low resolution images (blurry) to save memory usage. The black grid boundary lines do not exists in *SurfaceSlide*; it is drawn here for illustration purpose only.

The 3×4 grid was then extended into a large 9×12 grid (3072×2304 pixels) to not only accommodate these 12 on-screen image blocks, but also to cache an additional 96 neighbouring blocks as shown in Figure 8. When the on-screen FoV is panned, the maximum amount of movement in the horizontal direction is a screen width of 1024 pixels, whereas for panning vertically, the maximum amount of movement is 768 pixels. The 9×12 grid is sufficient to accommodate any single panning operation without introducing white backgrounds. Zooming operations also benefit from this caching approach. When zoom-in is performed, the on-screen FoV enlarges and becomes blurred. The blurred image is then replaced by its sharp counterparts by loading a 3×4 grid of high resolution images from the server. When zoom-out is performed and all grids are moving towards the centre of the screen, there is a good chance that the 9×12 grid is still large enough and will not bring in white background regions. The whole grid will only need to be reloaded from the server if any part of the 9×12 grid moves out of the screen, rather than after each single affine transform.

To load a large 9×12 grid of 256×256 pixel blocks consumes a considerable physical memory space and requires a significant downloading time. However, we have designed the system to load down-sampled low resolution images using interpolation. For an example, if the current FoV is at 40× magnification, and a 128×128 pixel block is loaded at 20× magnification then displayed (enlarged) in its corresponding 256×256 pixel grid, only 25% of the image data is actually loaded comparing to the loading a 256×256 pixel block at 40× magnification. This results in conservation of memory usage as well as reduction of downloading time. To preserve image quality, the centre 3×4 grid is loaded at 100% resolution, whereas the remaining 96 grids load only down-sampled low resolution images. The further a grid is sitting from the centre, the more blurred it becomes (Figure 8). The scaling factors used for down-sampling for all grids are listed in Table 2. We compared the latter to the amount of image data (in pixels) accrued by loading 9×12 grid of 256×256 pixel blocks at full resolution. Our approach loads 75.58% less image data and therefore reduces memory usage significantly and also image downloading time.

**Table 2:** The image size of each grid (% of full resolution image blocks).

		Column of the grid (% of image block sizes)											
		1	2	3	4	5	6	7	8	9	10	11	12
	**1**	6.25	12.5	12.5	12.5	12.5	12.5	12.5	12.5	12.5	12.5	12.5	6.25
	**2**	6.25	12.5	12.5	12.5	12.5	12.5	12.5	12.5	12.5	12.5	12.5	6.25
	**3**	6.25	12.5	25	25	25	25	25	25	25	25	12.5	6.25
**Row of the grid**	**4**	6.25	12.5	25	25	100	100	100	100	25	25	12.5	6.25
**(% of image**	**5**	6.25	12.5	25	25	100	100	100	100	25	25	12.5	6.25
**block sizes)**	**6**	6.25	12.5	25	25	100	100	100	100	25	25	12.5	6.25
	**7**	6.25	12.5	25	25	25	25	25	25	25	25	12.5	6.25
	**8**	6.25	12.5	12.5	12.5	12.5	12.5	12.5	12.5	12.5	12.5	12.5	6.25
	**9**	6.25	12.5	12.5	12.5	12.5	12.5	12.5	12.5	12.5	12.5	12.5	6.25

When a digital slide is panned or scaled, if down-sampled grids enter the screen area, their full resolution counterpart will be loaded to replace the blurred version, whereas the rest of the grid contents stay unchanged. In this way, a much smoother image transaction is achieved using smart caching.

### *D*. D. Usability Study

To find out the advantages and disadvantages of using multitouch technology in digital pathology and the robustness of the developed *SurfaceSlide* platform, a usability study was designed to compare *SurfaceSlide* with two other commercially available digital slide viewing and annotation applications, which are primarily mouse and keyboard controlled applications: Aperio ImageScope and the PathXL online viewer. Although the main objective of the usability study was to compare the novel multitouch approach with traditional mouse-keyboard based methods, readers should be aware of the rapid development of multitouch based applications in digital pathology over the last few months (in 2011), such as the Multitouch Microscope [30], SlidePath Gateway [31] and WholeSlide [32] apps for i-Pad, as well as the i-Pad slide viewer developed by our group with PathXL Ltd [33].

Thirteen people (9 males and 4 females) participated in the usability study, among them 11 are tissue imaging experts from the Department of Bio-Informatics and Bio-Imaging, Queen's University Belfast, and 2 are clinical pathologists from the Royal Victoria Hospital, Belfast. Participants performed an identical set of digital slide manipulation tasks using the same digital slide on the two mouse/keyboard driven platforms and the multitouch *SurfaceSlide* platform. Qualitative data was then gathered using questionnaires.

**Figure 9:**
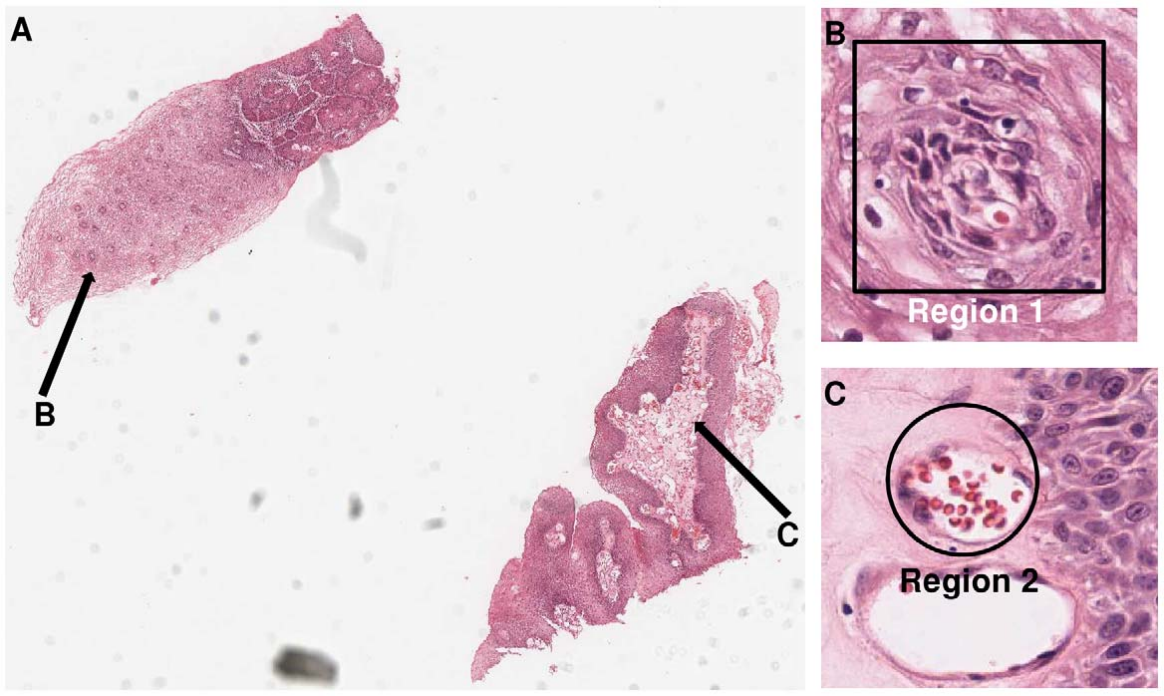
The tasks for the usability study. Participants were asked to view the cervical tissue digital slide on the left hand side (26,791×21,039 pixels). They were then asked to perform a number of panning and zooming operations in order to identify the two small regions (Figure B & C) from Figure A at a high magnification, and subsequently to annotate these two regions with either a rectangle or a circle, accompanied with text input of either “Region 1” or “Region 2”.

The digital slide manipulation task is described in Figure 9. Participants were asked to locate and annotate two small regions at a high magnification using a number of panning and zooming operations. For Aperio ImageScope, a local digital slide was used, whereas for both *SurfaceSlide* and PathXL, the digital slide was downloaded remotely from a centralised server at runtime. The questionnaire asked participants to rate nine questions which were related to different aspects of usability, and these questions are listed in Table 3. For each of the nine questions, users were asked to tick a value from a discrete scale between 1 and 10, where the choice of 1 indicates the best and the choice of 10 indicates the worst. Finally every participant was given the option to write down additional comments.

**Table 3:** The questions presented in the usability study questionnaire.

	Questions
**1**	How fun or boring it is to use each of the 3 platforms?
**2**	How easy or difficult it is to navigate images (to move image horizontally, vertically, zoom in/out)?
**3**	How easy or difficult it is to find where the menu is, and how easy it is to use it?
**4**	How easy or difficult it is to draw a circle and a rectangle annotation?
**5**	How easy or difficult it is to type in a text annotation?
**6**	How sharp or poor are the quality of images?
**7**	How slow or fast the system reacts after user commands?
**8**	How slow or fast it is to locate an area of interest?
**9**	What is your recommendation to use the 3 systems? (highly recommended/not recommended)

To ensure that the usability study was as equitable as possible across participants, equipments and samples were carefully chosen. First of all, high screen resolution was enforced. The screen resolution of Microsoft Surface is set at 1024×768 pixels, which cannot be improved without altering the hardware. When setting up the Aperio ImageScope and the PathXL online viewer, the high screen resolution of 1920×1200 pixels was set to ensure the best image quality with the use of ATI Radeon HD 2400 Pro graphic card and a 24 inch Dell U2410 monitor with a high performance Dell Optiplex 760 computer. Secondly, the *SurfaceSlide* and PathXL online viewer shared the same high network speed of 1.0 Gbps. Because a local digital slide was used for testing Aperio ImageScope, Internet connection was not required. Thirdly, an identical cervical histological digital slide was used across the three test platform. This slide was hematoxylin and eosin (H&E) stained, and scanned at 40× magnification with JPEG compression at the compression quality of 30. The entire size of the slide was 26791×21039 pixels. Finally, to avoid some participants remembering where the ROIs were, this was balanced out by allowing participants to use any of the three platforms in any random order.

## Results

Digital pathology and virtual microscopy has expanded rapidly over the last decade with the introduction of digitally scanned glass slides from cytology and histology. However, to date, most studies and manufacturers of slide scanners have focused their attentions on the slide scanning process and in digital slide analysis. A small number of studies have been undertaken to investigate human–digital slide interaction and to address the challenges arising from the ergonomics of digital slide viewing. With the commercial availability of multitouch technology, it is now possible to introduce novel interaction methods for pathology. This study used Microsoft Surface, a tabletop multitouch computing platform, for the development of *SurfaceSlide* and the investigation of a number of technical issues in the design of multitouch and the usability of multitouch in pathology. Microsoft Surface is an ideal candidate platform for the rapid prototyping of multitouch digital slide manipulation applications, which is enhanced by a set of software packages supported by Microsoft, such as the Surface SDK, Visual Studio, WPF and XMAL.

Using *SurfaceSlide*, users are able to navigate (to pan, zoom in and out) digital slides using multiple finger touches. Users are also able to annotate slides using rectangular and circular shapes to highlight regions of diagnostic interest. Free style text input is enabled as part of annotations.

To minimise the delay of transferring digital slide image data from remote image server, we developed a smart caching module. A large 9×12 grid was created with each grid element capable of holding a 256×256 pixel image block. To save memory usage and downloading time, boundary grids use down-sampled images, which save 75.58% of memory usage when compared to loading high resolution images for all of the grids. The resultant digital viewing experience is smooth and comparable with the viewing of local image data.

**Figure 10:**
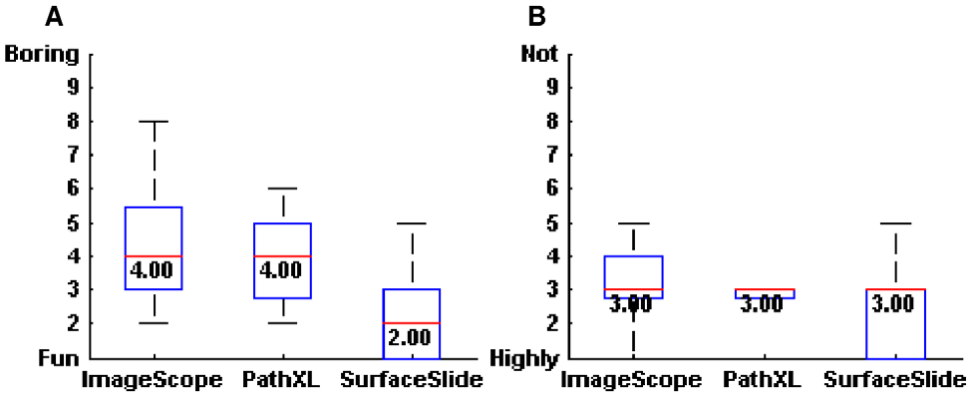
Box plot results of the qualitative ratings for three platforms. (A) Box plot illustrating the level of enjoyment participants reported when using each platform, (B) Box plot of user recommendations for each of the three platforms. *Please note that the smaller the value on the y-axes the better the results.

We undertook a usability study to compare *SurfaceSlide* with traditional mouse-keyboard methods. Due to the small number of participants, dependency measures were not conducted. In terms of rating for the level of “fun” across the three platforms, *SurfaceSlide* achieved the best score (median = 2, mode = 1,2). *SurfaceSlide* also achieved the best score for the rating of user recommendation (median = 3, mode = 1,3). These results are shown in Figure 10. The novelty of multitouch, and the intuitive and fun way in which individuals can interact with pathology images certainly encourages interest in this technology.

**Figure 11:**
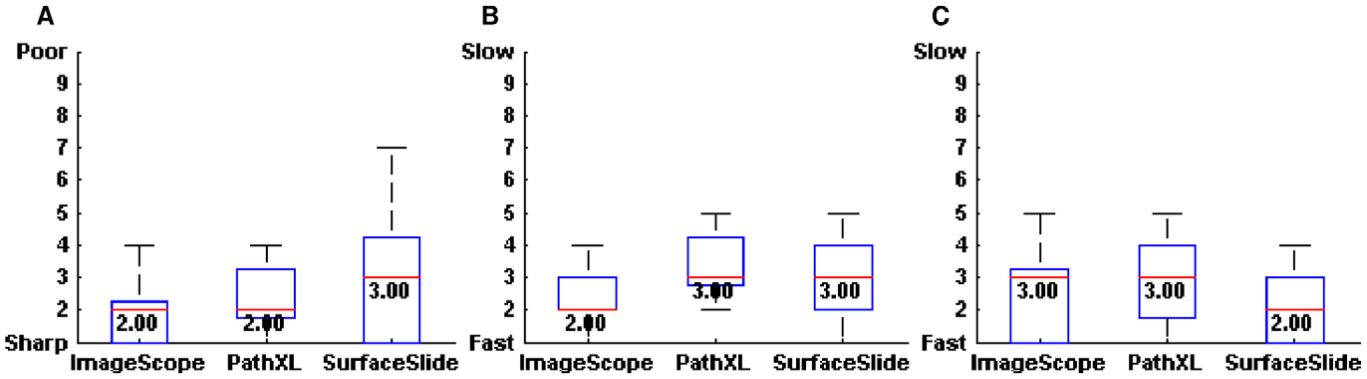
Box plot results of the subjective usability ratings for three platforms (continued). (A) Box plot of perceived digital slide image quality, (B) Box plot illustrating the perceived system reaction speed for each platform, (C) Box plot illustrating the perceived speed of location of ROIs. *Please note that the smaller the value on the y-axes the better the results.

In terms of the image qualities of digital slides, *SurfaceSlide* rated the worst (median = 3), however its mode value (mode = 1), the most common response, suggested that the image quality of *SurfaceSlide* was perceived to be the sharpest (Figure 11A). This confusion of *SurfaceSlide* image qualities could be the result of low resolution of the Microsoft Surface screen (1024×768 pixels) and the use of the back-projection monitor. However, the next generation Microsoft Surface 2 (Samsung SUR40) utilises 1920×1080 pixel high resolution 40 inch LCD screen and produces crystal clear image quality. Therefore, the concern with image qualities on *SurfaceSlide* could be easily solved by porting the software code onto Microsoft Surface 2.

Figure 11B indicates that ImageScope produced the fastest reaction after an instruction was issued by the user. This was attributable to the fact that ImageScope was accessing local imageries whereas the other two platforms were accessing the image across the Internet at runtime.

*SurfaceSlide* was the fastest system to locate ROIs (Figure 11C). The multitouch experience provides the unique capability of panning and zooming simultaneously on digital slides, which is currently not possible with mouse driven interfaces. This is likely to be the underlying reason why subjects thought *SurfaceSlide* was the fastest system to locate ROIs.

Usability perception of the difficulty to perform four types of tasks is given in Table 4. These four types of tasks were i) Image navigation, ii) finding and using the on-screen menu, iii) drawing a circle and a rectangle, and iv) typing texts as annotations. *SurfaceSlide* scored the best or equal best for all four tests. Establishing a brand new user interface, the NUI, it was expected that some participants would perceive certain difficulties. However, as suggested by the results listed in Table 4, *SurfaceSlide* scored very well by comparison to traditional GUI (Graphical User Interface) based platforms. It compared favourably with the advantages of touchable user interface that have been shown in other studies [23], [34]. It leverages our well developed skills for the manipulation of objects, and facilitates user-object interactions by direct contact. Even without training, users already “know” how to view and annotate digital slides. It is also noticeable that users touch *SurfaceSlide* in a slightly different way. When panning a slide, some users use a single finger whereas others use five fingers. When scaling a slide, some user uses two single fingers (from a single hand, or one finger from each hand) whereas others use all the ten fingers from two hands. As Microsoft Surface is able to recognise 52 simultaneous touches, this difference in user gestures (the number of fingers applied) does not affect the operations users are trying to perform.

**Table 4:** Usability ratings for how easy or difficult performing certain tasks was using three different platforms (median values).

	ImageScope	PathXL	*SurfaceSlide*
**Image navigation**	2	3	2
**To use the menu**	4	3	2
**To draw shapes**	4	3	3
**To type in texts**	4	2	2

Extra comments were given from the usability experiment participants, especially the following comment from one of the two clinical pathologists. The pathologist mentioned that the resolution of *SurfaceSlide* was not as good as the other two platforms, but still good. This could lead to future studies in investigating the factors affecting user viewing experiences and diagnostic accuracy, as well as in defining possible future criteria in digital pathology quality control.

## Discussion

*SurfaceSlide*, utilising multitouch technology was developed in this study. The usability experiment demonstrated that multitouch and smart caching approach, represent the most intuitive interface for human–digital slide interaction. Besides its ergonomic aspect, *SurfaceSlide* considered usability, reliability and speed. Speed of navigation is essential for diagnostic interpretation of images and has been an issue with mouse/keyboard driven interfaces. Diagnostic interpretation requires the ability to rapidly review slide at a variety of magnifications in tandem with simultaneous x, y navigation. This is made possible using multitouch technology, and we have demonstrated in this study that this speeds up the slide manipulation and viewing. Thus, the biggest issue for the Microsoft Surface and *SurfaceSlide* is the image quality which could be addressed with the new version of Microsoft Surface 2 (Samsung SUR40).

For the navigation of digital slides, *SurfaceSlide* allows the user to perform panning in any direction and to use simple finger touches. However most digital slide viewing systems [2], such as the Aperio ImageScope and the Hamamatsu NDP.view, can only perform the same task by holding down the left mouse button and moving the mouse simultaneously in any direction. *SurfaceSlide* certainly overcame the limitation that traditional microscopes are only able to move a glass slide either horizontally or vertically at one time. For the zooming of slides, the traditional microscope performs zooming by physically changing objective lenses. Hamamatsu NDP.view simulates this process by providing popular discrete low to high magnifications, such as 5×, 10×, 20× and 40× magnifications, whereas other viewers, such as Aperio ImageScope and PathXL viewer, are able to zoom in and out at the magnifications where physical objective lenses cannot achieve, such as 11× magnification, providing a smooth transaction during zooming operations. Neither conventional microscopy nor commercial digital slide viewers can perform zooming and panning operations simultaneously. *SurfaceSlide* is, in addition, able to zoom in and out at any arbitrary magnifications at any location of the current FoV. No other system has this capability. *SurfaceSlide* enables users to perform panning and zooming simultaneously to locate ROIs rapidly.

For the smart caching of digital slide, we introduced a simple approach which employs the delivery of image information in the surrounding regions of FoVs, specifically for the reduction of manipulation latencies. We acknowledge the existence of other robust methods which could speedup the online delivery of digital slides, such as cloud computing [35], [36] and novel image format (for fast decompression) [19], [37], [38]. However, this was beyond the scope of this study.

This study suggests that *SurfaceSlide*, utilising multitouch and smart caching for the manipulation of online digital slides, is a novel, practical and effective platform for digital pathology. It will certainly encourage the transfer of digital pathology into pathological education and routine diagnostics, and also facilitate the navigation and annotation of digital slides. We propose to explore the use of *SurfaceSlide* in an educational environment; either within a dedicated classroom or in a social environment, where students can easily access pathology based learning materials on a table top display. This study also takes us closer to the wider objective of providing an integrated highly functional multitouch environment for large image viewing in pathology and will almost certainly have implications for the wider application of this technology across other modalities in medical imaging.

As one of the first multitouch tools for pathology, *SurfaceSlide* still requires improvement to the following functions: i) Orientation: currently texts displayed on screen can only face one direction. ii) Multi-user: When multiple users are engaged in the viewing of one digital slide using one or more set of *SurfaceSlide*, proper user-control schemes are needed to allow the control of one user at a time.

Future work is needed to investigate the impact of the choice of digital platforms on clinical decision making process and diagnostic outcomes. This needs to be a carefully designed dedicated study with well defined specific diagnostic problems. Future study is also required to investigate the impact of displaying intermediate magnifications (e.g. 11× magnification) and the effects of using interpolation to achieve this with respect to image quality. *SurfaceSlide*, as well as Aperio ImageScope and the PathXL online viewer, all organise digital slide images in a pyramid structure and output intermediate magnification images using some sort of interpolation. However, there have not been any studies evaluating the impact of interpolation on the quality of images, and its influence on diagnostic and clinical decision making.

We conclude that as multitouch technology develops rapidly, with the release of handheld devices such as the Apple's i-Pad, the opportunities for developing novel image viewing and navigation tools in pathology will escalate. The basic concepts presented in this study can certainly translate to other devices when they become available. The authors fully expect that multitouch will gradually become the interface of choice for digital slide viewing on a range of table-top, vertical screen and handheld devices, representing an exiting change in how digital pathology will be delivered over the next few years.

### Availability

The Microsoft Surface machine, Surface SDK and Windows Vista operating system can be purchased from Microsoft. The source code for *SurfaceSlide* is publicly available at http://code.google.com/p/surfaceslide-yinhaiwang/using SVN checkout. Please note the source code is specifically designed for Microsoft Surface, it will NOT run on any personal computers (PCs). Licensing agreements regarding digital slide hosting and PathXL i-Server interface API can be obtained from PathXL Ltd. (www.pathxl.com).
